# Carbon Nanotube-Supported Bimetallic Core–Shell (M@Pd/CNT (M: Zn, Mn, Ag, Co, V, Ni)) Cathode Catalysts for H_2_O_2_ Fuel Cells

**DOI:** 10.1021/acsomega.3c05531

**Published:** 2023-10-02

**Authors:** Burak Yapici, Ozlem Gokdogan Sahin

**Affiliations:** Chemical Engineering Department, Konya Technical University, 42250 Konya, Turkey

## Abstract

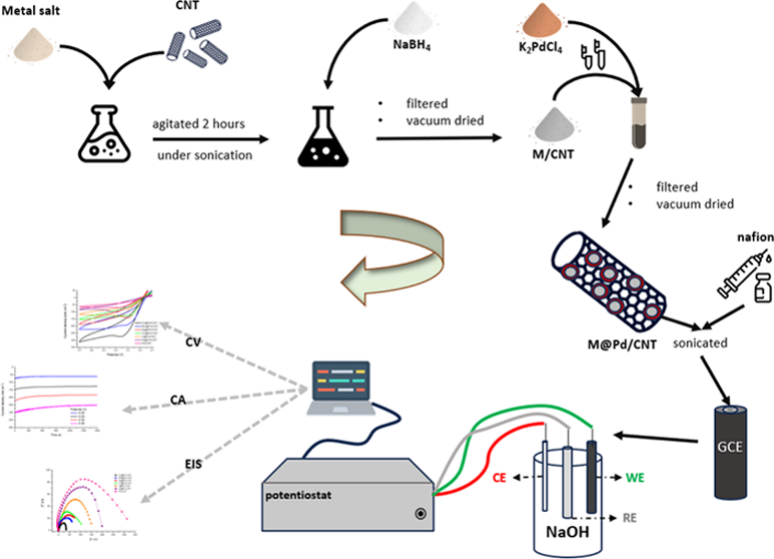

M@Pd/CNT (M: Zn, Mn, Ag, Co, V, Ni) core–shell and Pd/CNT nanoparticles were prepared by sodium borohydride reduction and explored as cathode catalysts for the hydrogen peroxide reduction reaction. Electrochemical and physical characterization techniques are applied to explore the characteristics of the produced electrocatalysts. The cyclic voltammetry (CV) experiments show that Zn@Pd/CNT-modified electrodes have a current density of 273.2 mA cm^–2^, which is 3.95 times higher than that of Pd/CNT. According to the chronoamperometric curves, Zn@Pd/CNT has the highest steady-state current density for the H_2_O_2_ electro-reduction process among the synthesized electrocatalysts. Moreover, electrochemical impedance spectroscopy (EIS) spectra confirmed the previous electrochemical results due to the lowest charge transfer resistance (35 Ω) with respect to other electrocatalysts.

## 1 Introduction

Fuel cells have gained much attention as effective and clean energy devices. They are emerging as prime energy transformation ways for several areas in fixed and mobile applications.^1^ However, price is a big barrier interrupting fuel cell distribution because of the requirement of a high loading of noble metals such as platinum.^2^ The cost can be reduced meaningfully by using alternative noble metals.^3,4^

Benchmarked with liquid fuels, hydrogen peroxide (H_2_O_2_) is a promising liquid fuel consisting of hydrogen and oxygen.^5^ H_2_O_2_ fuel cells have been receiving growing consideration due to their high energy density and cell potential. Hydrogen peroxide fuel cells have the benefits of no side products and, therefore, no poisoning of the modified electrodes.^6^ In addition, the H_2_O_2_ reduction reaction with two electrons has better kinetics than O_2_ reduction to H_2_O_2_ with four electrons.^7,8^ H_2_O_2_ can be used in a H_2_O_2_ fuel cell system in two ways, i.e., as a fuel or oxidant, and it can be constructed according to its usage.^9^

The superiority of H_2_O_2_ reduction has an outstanding influence on the execution of H_2_O_2_ fuel cell behaviors. Moreover, liquid H_2_O_2_ has the benefits of suitable carriage and storage for both space crafts and submarines. Concurrently, electro-reduction of H_2_O_2_ takes place in the two-phase boundary, which is more achievable than the reduction of O_2_ electro-reduction taking place in a three-phase zone.^10^ As a consequence of the above properties, H_2_O_2_ is an impressive substitute for O_2_ in fuel cells.^11^ The reduction of H_2_O_2_ is executed with the subsequent reaction (eq 1) in an alkaline medium
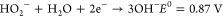
1Over the past few years, crucial attempts have been made to improve the electrode modification to improve the electron transfer effectiveness, which is extremely related to fuel cells’ performances. Among these above-mentioned options for the applications of fuel cells, electrode modification has a great effect on electron transfer.^12^

Currently, noble metals, transition metals, metal oxides, and metal and organic compound mixtures are the main electrode materials used in H_2_O_2_ reduction.^13−15^ Among these, Pd as the illustrative noble metal is usually taken into consideration to improve catalyst activity, since it has the same features as Pt, but it is more abundant and cheaper. Considering both metals present the same conformation,^9,16^ Pd can be used instead of Pt due to its high electron utilization rate and adsorption capacity.^17^ Moreover, Pd could encourage the breaking of O–O bonds in H_2_O_2_.^11^ However, the main drawback in the design of fuel cells is the cost of the precious metals used for catalyst manufacturing. This problem will be solved by developing high-performance and low-price electrode catalysts. Therefore, researchers have concentrated on adding less expensive metals into Pd catalysts to increase the catalytic activity and reduce the cost of Pd metal by exploiting the structure, electronic, and synergistic effects among different materials. Literature studies^7,15,16,18^ have confirmed that owing to synergistic catalytic outcomes, bimetallic catalysts express higher catalytic activity than any single one.^19^ According to research by Sun et al.,^20^ Cu in the PdCu/C electrode enters the Pd lattice and modifies its electronic structure, giving the hydrogen peroxide reduction reaction better performance than commercial Pd/C. For instance, because of the strong electronegativity of Au and the synergistic interaction between Pd and Au, the catalytic performance of the electro-reduction of H_2_O_2_ might be enhanced by doping the Pd electrode with a second metal of Au.^21^

Typically, the core–shell form has an efficient morphology for increasing catalytic activity. These nanoparticles are commonly preferred to alloy nanoparticles due to the improvement in the usage level of a noble metal at the external surface.^22^ Otherwise, the core and shell structure decreases the load of the metal and increases its impact.^23,24^ This structure can exhibit the effects of synergistic between the core and the shell that improves the electrochemical reaction activity.^25^ Besides, the activity of the core–shell structure is related to the underlying interface between the core and shell metals due to the bimetallic mechanism.

As noble metals employed in catalyst synthesis are expensive, support materials are used to moderate the cost. Catalysts are dispersed over the support materials rather than being used as one piece, reducing the load of the catalyst used and increasing the catalyst surface area.^26^ Besides that, the support materials used together with the catalyst can enhance the catalytic activity.^27^ Carbon-supported materials such as graphene, graphene oxides, and carbon nanotubes have higher activity and stability than unsupported metal catalysts.^28^ With their benefits of a high specific surface area, good chemical stability, low resistance, thermal stability, great flexibility, and superior electrical properties, carbon nanotubes (CNTs) are a common choice for electrode materials.^29^

In this paper, the NaBH_4_ reduction method was used to create M@Pd/CNT (M: Zn, Mn, Ag, Co, V, Ni) and Pd/CNT. The structural, morphological, and electrochemical features of the prepared materials were characterized. Using a three-electrode setup, the behavior of the catalysts regarding the hydrogen peroxide reduction reaction was studied with electrochemical procedures. Also, the impact of experimental conditions on the catalytic activity of electrocatalysts was evaluated.

## 2 Experimental Section

### 2.1 Apparatus

A CHI660E potentiostat device was used for electrochemical measurements. The electrode cleaning and dispersion processes by adding Nafion to catalyst powders were carried out in a Branson 1510-MTH ultrasonic bath. A Scilogex MS–H-S was used as a magnetic stirrer.

### 2.2 Chemicals

Sodium hydroxide (NaOH) and hydrogen peroxide (H_2_O_2_) were used in the experimental studies. Ethanol (C_2_H_5_OH) and alumina powder (Al_2_O_3_) were used to clean the working electrodes. In the catalyst preparation stage, palladium(II) chloride (PdCl_2_), manganese(II) chloride (MnCl_2_), silver nitrate (AgNO_3_), cobalt chloride (CoCl_2_), zinc chloride (ZnCl_2_), vanadium(V) oxide (V_2_O_5_), nickel(II) sulfate (NiSO_4_), sodium borohydride (NaBH_4_), carbon nanotubes (CNT), and Nafion solution were used and obtained from Alfa Aesar.

### 2.3 Catalyst Preparation

Bimetallic core–shell catalysts with 10% Pd content were obtained using the NaBH_4_ reduction technique. In order to synthesize M/CNT, an appropriate quantity of ZnCl_2_, MnCl_2_, AgNO_3_, CoCl_2_, VCl_3_, and NiSO_4_ solutions was prepared in water first. The solution was then agitated for 2 h while the CNT support material was added under sonication. A certain quantity of NaBH_4_ was mixed to obtain M/CNT nanoparticles. Then, it was filtered, cleaned, and vacuum-dried at 90 °C for 14 h. A suitable quantity of M/CNT nanoparticles and K_2_PdCl_4_ was added to water to synthesize bimetallic M@Pd/CNT (M: Zn, Mn, Ag, Co, V, Ni) catalysts.

### 2.4 Catalyst Characterization

M@Pd/CNT and Pd/CNT nanoparticles were characterized by using X-ray diffraction (XRD) investigations. The crystallographic structure of these catalysts was examined with XRD. The particle crystallite size was obtained from Scherrer equality. The morphology and particle dimensions were examined by transmission electron microscopy (TEM) (Zeiss Sigma 300) operating at 200 kV. The Specs-Flex gadget used X-ray photoelectron spectroscopy (XPS) analysis to detect the oxidation status of the Zn@Pd/CNT catalyst.

### 2.5 Electrode Modification

The prepared solid catalyst was spread in Nafion solution. Catalyst inks were obtained by an ultrasonic bath for 30 min. 3 μL was taken from the catalyst ink with the help of a micropipette, then applied on the working electrode surface, and dried.

### 2.6 Electrochemical Analysis

Using a potentiostat (CHI660E) linked to a computer, cyclic voltammetry (CV), chronoamperometric (CA), and electrochemical impedance spectroscopy (EIS) experiments were conducted in an electrochemical cell with three electrodes such as Ag/AgCl reference, GCE working, and Pt wire counter electrodes. The working electrode was cleaned with alumina powder and ethanol after each measurement.

## 3 Results and Discussion

### 3.1 Physicochemical Characterization

**Figure 1:**
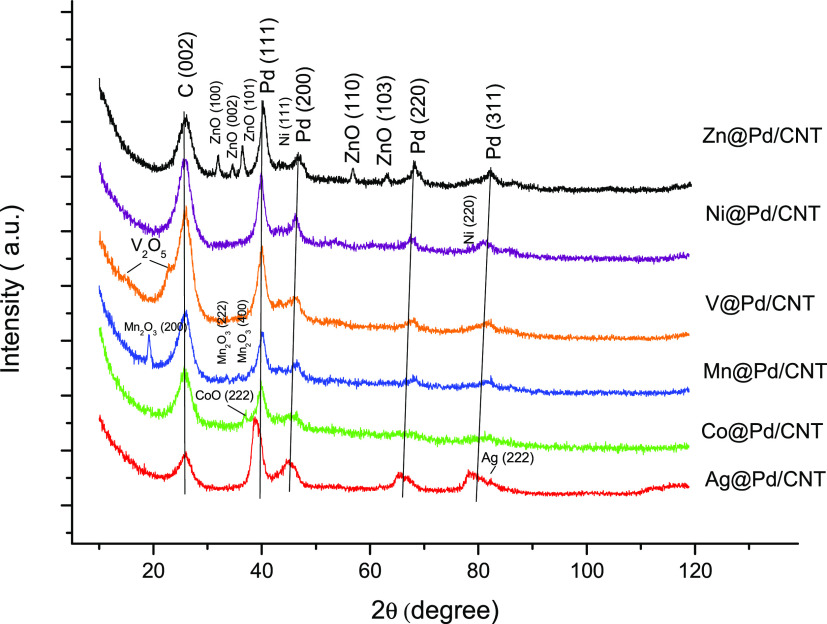
XRD graphs of the M@Pd/CNT nanoparticles.

The XRD spectra of M@Pd/CNT catalysts are listed in Figure 1. All of the catalysts showed a wide peak at about 26° relative to the hexagonal (002) carbon plane (JCPDS card no. 75-1621). Furthermore, the XRD findings of the catalysts revealed the (111), (200), (220), and (311) planes, confirming the Pd face-centered cubic (fcc) crystalline structure (JCPDS card no. 46-1043). Diffraction peaks appearing in the pattern of the Zn@Pd/CNT catalyst at 2θ values of 32.0, 33.2, 36.6, 40.26, 47.1, 56.7, 63.0, and 68.3° were assigned to ZnO(100), ZnO(002), ZnO(101), Pd(111), Pd(200), ZnO(110), ZnO(103), and Pd(220), respectively. The Co@Pd/CNT catalyst exhibited diffraction peaks at 36.9, 40.23, 46.6, 65.6, and 81.3° corresponding to the CoO(222), Pd(111), Pd(200), Pd(220), and Pd(311) planes, respectively. Furthermore, low-intensity PdO peaks were observed. The Mn@Pd/CNT catalyst’s diffraction peaks at 19, 31, 36, and 41° can be attributed to Mn_2_O_3_(200), Mn_2_O_3_(222), and Mn_2_O_3_(400) (JCPDS card no. 24-0508). Low-intensity PdO peaks were identified at 43.5, 51.2, and 60.2°. From the XRD analysis of V@Pd/CNT, VO_2_ and V_2_O_5_ peaks are observed, except for the Pd and PdO peaks. The diffraction peaks at 37.9, 43.4, 65.1, 65.9, 78.1, 79.4, and 81.7° of the Ag@Pd/CNT catalyst were attributed to Pd(111), Pd(200), Ag(220), Pd(220), Ag(311), Pd(311), and Ag(222), respectively (JCPDS card no. 04-0783). The XRD pattern of Ni@Pd/CNT showed (111) (44.2°), (200) (52.9°), and (220) (78.8°) peaks. The crystallite size of M@Pd/CNT (M: Zn, Mn, Co, Ag, V, Ni) catalysts was calculated to be 5.50, 5.40, 6.51, 6.52, 6.96, and 7.00 nm, respectively, using the Scherrer equation.

**Figure 2:**
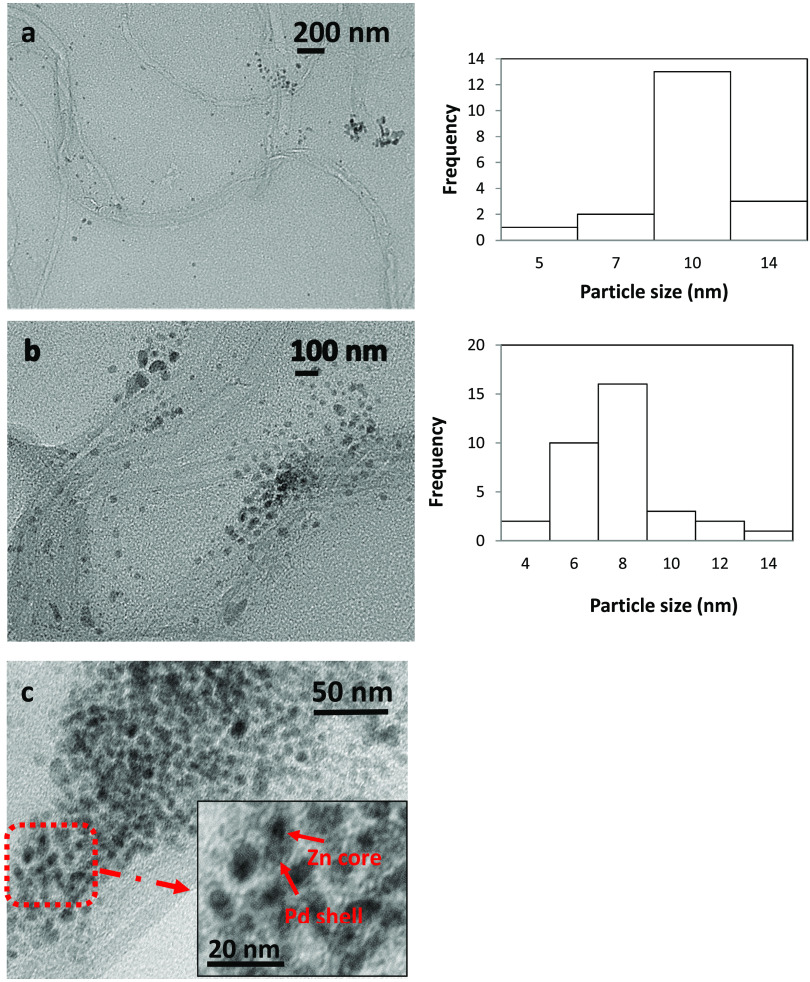
TEM images of the Zn@Pd/CNT catalyst at (a) 200, (b) 100, and (c) 50 and 20 nm.

The TEM analysis of the Zn@Pd/CNT catalyst at different scales and the graphs of particle size distribution are presented in Figure 2a,2b. The inset of Figure 2c shows that Zn nanoparticles have a black core and Pd nanoparticles have a bright shell, representing the formation of core–shell nanostructures. As seen in the figure, metal nanoparticles show a regular distribution on the CNTs. From the ImageJ program, the average particle size of Zn@Pd/CNT was found to be 7.7 nm.

**Figure 3:**
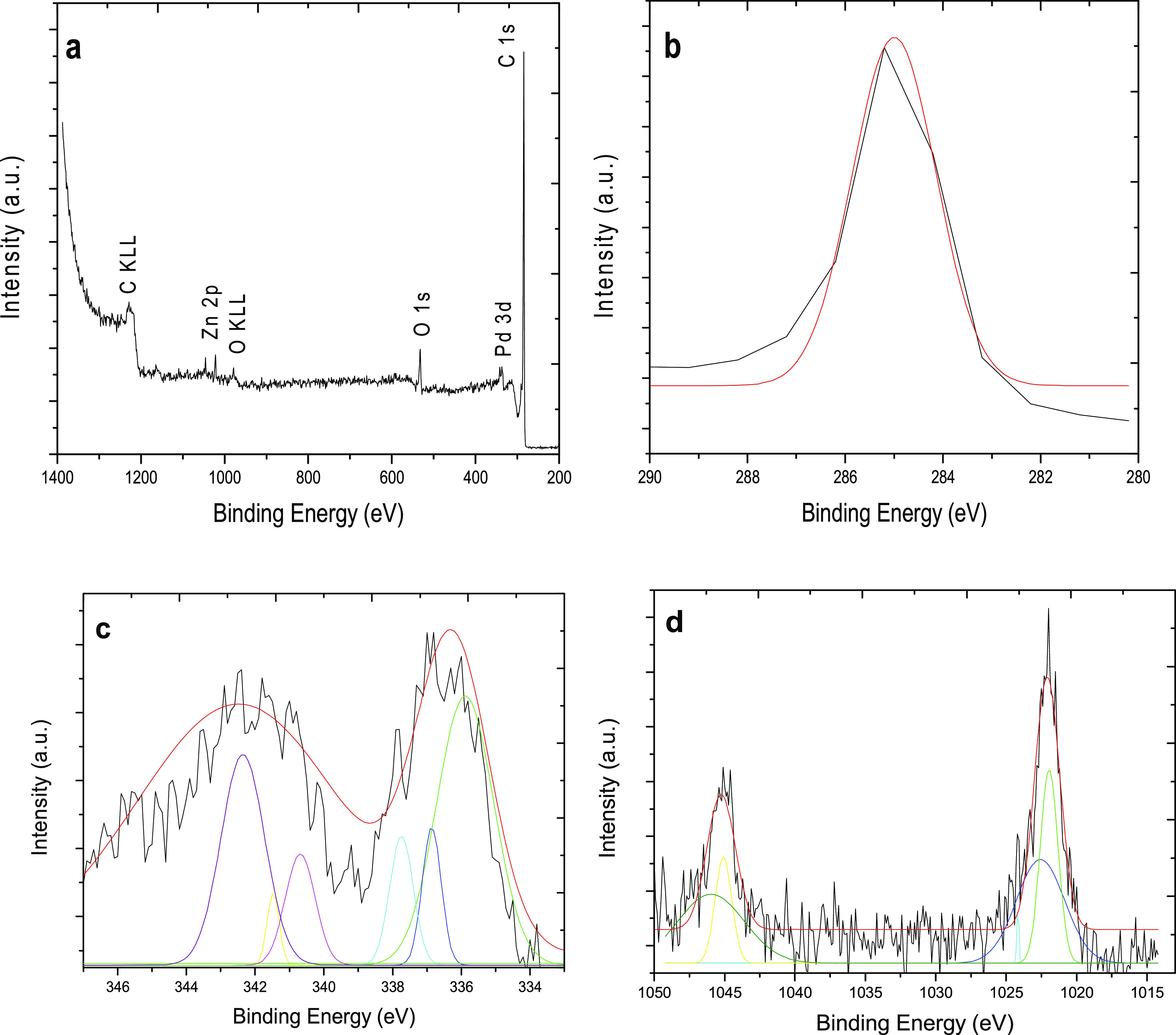
Spectrum of (a) general, (b) C 1s, (c) Pd 3d, and (d) Zn 2p of the Zn@Pd/CNT catalyst.

Following that, the oxidation states of the Zn@Pd/CNT catalyst were determined by XPS. Zn@Pd/CNT total spectra revealed C 1s, O 1s, Pd 3d, and Zn 2p peaks (Figure 3a). Figure 3b shows the binding energy of C 1s. The spectra of Pd 3d in Figure 3c could be ascribed to Pd^0^ (at 334.8 and 341.6 eV), PdO (at 342.2 eV), Pd(OH)_*x*_ (at 336.4 and 340.9 eV), and PdO_2_ (at 337.6 eV). Furthermore, the spectra of Zn 2p at 1022.1 and 1045.2 eV demonstrated that Zn was mostly present as Zn^2+^ (Figure 3d). In addition, the uncurved peak at 1023.1 eV is considered to be Zn(OH)_2_. The Zn@Pd core–shell had a greater Pd concentration, suggesting that Pd was mostly dispersed in the outer layer shell.

### 3.2 Electrochemical Characterization

**Figure 4:**
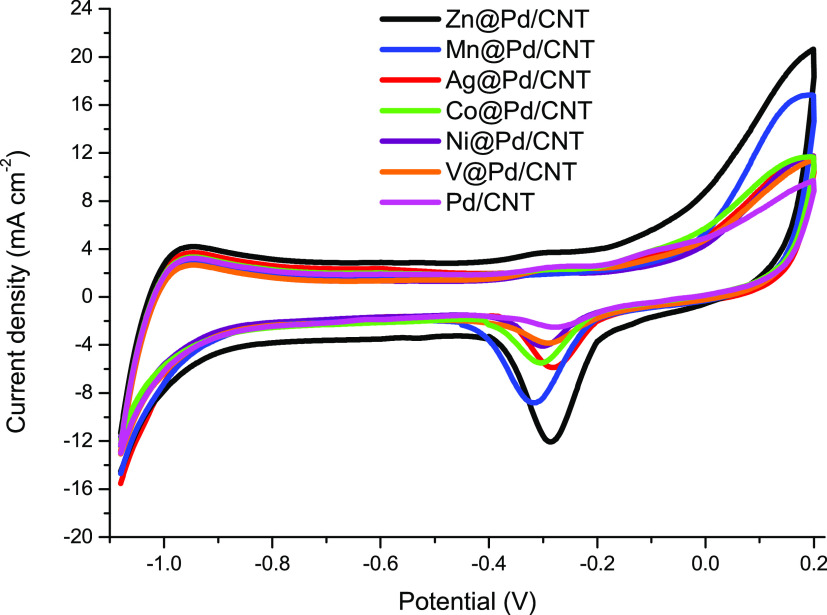
Cyclic voltammograms of M@Pd/CNT (M: Zn, Mn, Ag, Co, V, and Ni) and Pd/CNT-modified GCEs in 1.0 M NaOH at 20 mV s^–1^.

Figure 4 shows the CV curves of M@Pd/CNT (M: Zn, Mn, Ag, Co, V, and Ni) and Pd/CNT-modified glassy carbon electrodes in a 1.0 M N_2_-saturated sodium hydroxide electrolyte solution. The modified electrodes have shown the common behavior of palladium-based catalysts in an alkaline medium.^30−32^ The peaks between −1.1 and −0.6 V are attributed to the hydrogen adsorption/desorption on palladium, while the prominent cathodic peaks typical of palladium oxides are between −0.45 and −0.05 V in the back scan, which is generally employed to calculate the catalysts’ electrochemical surface areas (ECSAs, eq 2)
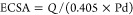
2where *Q* is the consumed charge for PdO reduction on the modified electrode, and the needed charge for PdO reduction is 0.405 mC cm^–2^.^23,31,33^ The ECSA values of Zn@Pd/CNT (22.1 m^2^ g^–1^) are 4.70, 3.81, 2.95, 1.88, 1.65, and 1.12 times higher than Pd/CNT (4.7 m^2^ g^–1^), Ni@Pd/CNT (5.8 m^2^ g^–1^), V@Pd/CNT (7.5 m^2^ g^–1^), Co@Pd/CNT (11.6 m^2^ g^–1^), Ag@Pd/CNT (13.4 m^2^ g^–1^), and Mn@Pd/CNT (19.8 m^2^ g^–1^), respectively. The greater area for Zn@Pd/CNT supports improved electrochemical activity and Pd consumption in an alkaline medium compared to other produced catalysts.

The cyclic voltammograms of M@Pd/CNT (M: Zn, Mn, Ag, Co, V, Ni) and Pd/CNT-modified GCE were obtained in the potentials varying from −0.1 to −0.7 V. Without H_2_O_2_, no recognizable change in current was detected over the full potential range, indicating that no distinguishable activity occurred on the electrode surface. However, the cathodic scan at around −0.3 V, which represents the H_2_O_2_ reduction that follows a two-proton and two-electron reaction, showed a substantial rise in the response after the H_2_O_2_ addition to the solution (eq 3).^34^
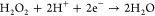
3

**Figure 5:**
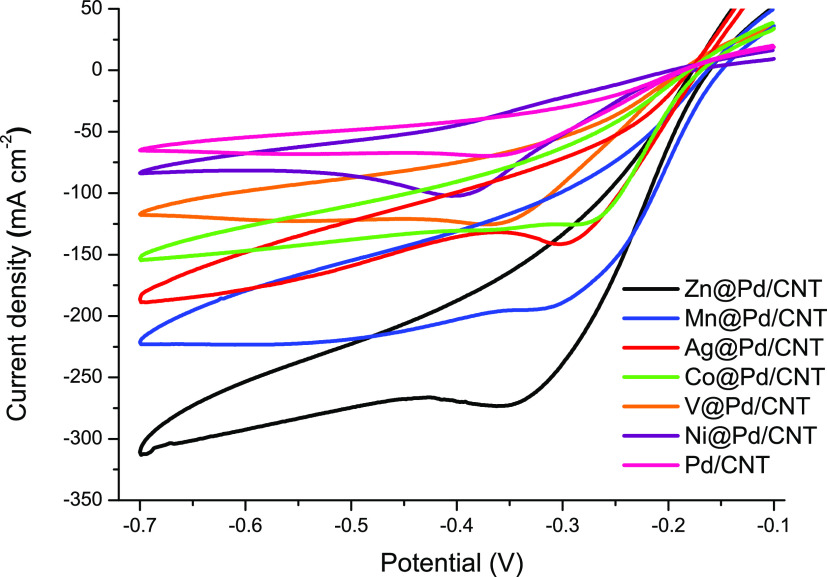
Cyclic voltammograms of M@Pd/CNT- (M: Zn, Mn, Ag, Co, V, Ni) and Pd/CNT-modified GCEs in 0.25 M H_2_O_2_ + 1.0 M NaOH at 20 mV s ^–1^.

As shown in Figure 5, the current density of hydrogen peroxide reduction on Zn@Pd/CNT (273.2 mA cm^–2^) is 3.95, 2.68, 2.27, 2.18, 1.93, and 1.41 times higher than those on Pd/CNT (69.2 mA cm^–2^), Ni@Pd/CNT (102.1 mA cm^–2^), V@Pd/CNT (120.3 mA cm^–2^), Co@Pd/CNT (125.6 mA cm^–2^), Ag@Pd/CNT (141.5 mA cm^–2^), and Mn@Pd/CNT (193.4 mA cm^–2^), respectively. Core metals (Zn, Mn, Ag, Co, V, Ni) coupling with palladium atoms can change the outer electronic structure of palladium, which affects the energy involved in the adsorption/desorption of palladium to H_2_O_2_. In other words, the lattice strain and synergistic effects in the core–shell region can increase the utilization of metal atoms and result in changes in the electronic structure, which can significantly improve the catalyst activity and cause differences in the current densities. Among the catalysts prepared, the Zn@Pd/CNT catalyst showed the best activity in the H_2_O_2_ electro-reduction reaction with the highest peak current density. The consequences confirmed that the modification of GCE with highly conductive Zn@Pd/CNT modification to the GCE increased the electrode conduction and significantly improved the conductivity of the electrode. Subsequent experiments were carried out with the Zn@Pd/CNT catalyst.

**Figure 6:**
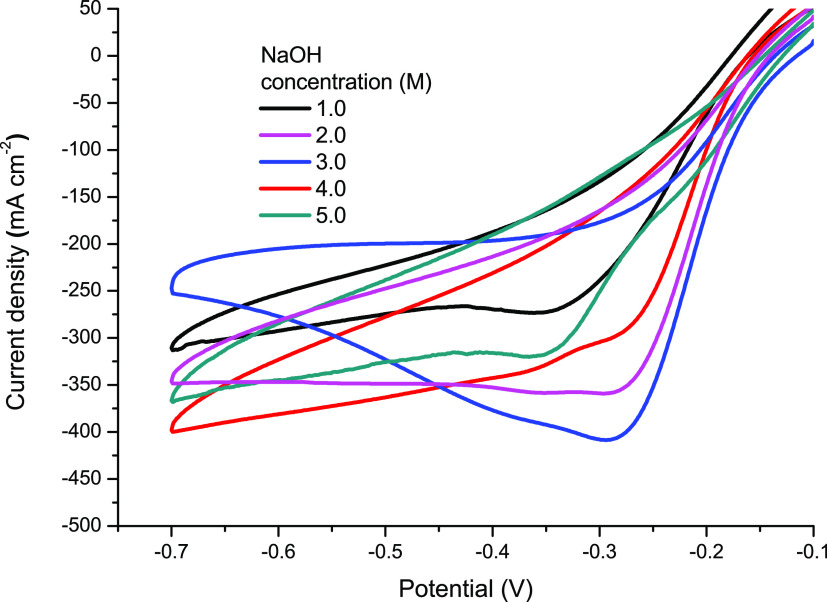
Cyclic voltammograms of Zn@Pd/CNT-modified GCEs at different concentrations (1.0–5.0 M) of sodium hydroxide and 0.25 M hydrogen peroxide.

The electro-reduction of H_2_O_2_ on the Zn@Pd/CNT-modified GCE has been investigated for different experimental conditions. First, the effect of the NaOH concentration was studied by cyclic voltammetry for various concentrations of NaOH and a 0.25 M H_2_O_2_ solution. Clearly, the NaOH concentration effect for H_2_O_2_ electro-reduction on the Zn@Pd/CNT-modified GCE is much more significant, as shown in Figure 6. The H_2_O_2_ electro-reduction current increases as the NaOH concentration increases up to 3.0 M.^3,35^ The current density then decreases with a further increase in NaOH molarity until 5.0 M.^36^ The increase in the NaOH concentration has no advantage for the improvement of hydrogen peroxide reduction; therefore, it is useless, and as a result, the optimum NaOH value is 3.0 M.

**Figure 7:**
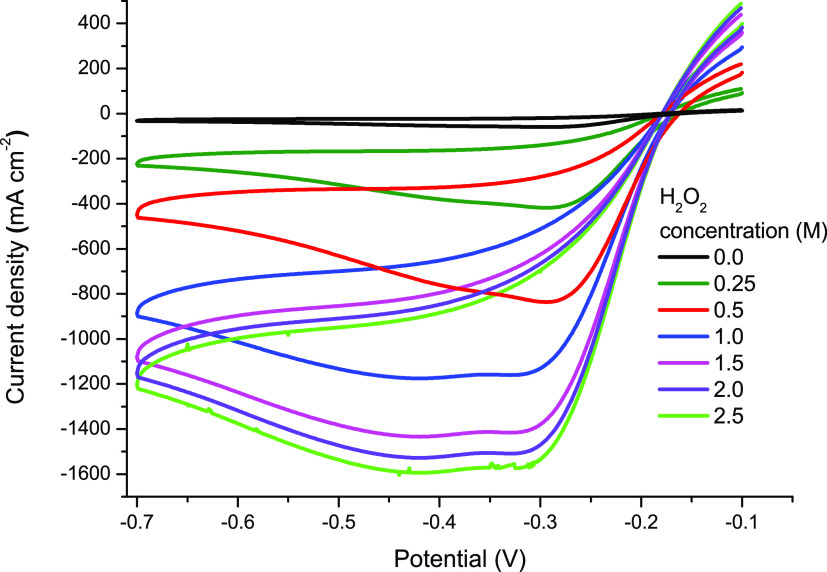
Cyclic voltammograms recorded on Zn@Pd/CNT-modified GCE in a 3.0 M NaOH solution at different concentrations of H_2_O_2_ (0.0–2.5 M).

The effect of H_2_O_2_ concentration on H_2_O_2_ electro-reduction was investigated with Zn@Pd/CNT-modified GCE. The cyclic voltammetric results of different concentrations of H_2_O_2_ are shown in Figure 7. Similar to NaOH, the electro-reduction current density on Zn@Pd/CNT-modified GCE also shows a remarkable increase with the change in H_2_O_2_ concentration. The peak current does not significantly increase when the H_2_O_2_ concentration is increased further, though. Given that H_2_O_2_ is known to be unstable in strong alkaline electrolytes, this tendency is likely caused by severe H_2_O_2_ chemical breakdown.^3,30^

Zn@Pd/CNT is an excellent catalyst for the reduction of hydrogen peroxide by comparison with other catalysts synthesized for the reduction of H_2_O_2_ in the literature.^8,14,30,37,38^ By the evaluation of H_2_O_2_ electro-reduction on different modified electrodes in Table 1, the prepared Zn@Pd/CNT cathode catalyst displays a lower reduction potential and a higher current density than other reported cathode catalysts for H_2_O_2_ reduction.

**Table 1:** Comparison of Different Catalysts for Electrochemical Reduction of H_2_O_2_

catalysts	electrolyte concentration NaOH (M)	H_2_O_2_ concentration (M)	potential (V)	current density (mA cm^–2^)	reference
PdCoMn_2_O_4_	3.0	0.7	– 0.80	580	(8)
Pd@Co_3_O_4_/Ti	3.0 (KOH)	0.4	– 0.40	145.8	(14)
Pd–Co_3_O_4_/ RGO@PI	2.0	0.5	– 0.80	962	(17)
Pd/rGO/NF	3.0	0.5	– 0.50	303	(30)
Pd@SnO_2_/Ni	3.0	0.5	– 0.54	320	(37)
PRN (Pd/rGO/Ni)	2.0	0.5	– 0.60	450	(38)
Pd-CNT/Ni foam	3.0 (KOH)	0.4	– 0.80	323	(39)
CoPd@graphite	1.0	1.4	– 0.60	270	(40)
Co/Co_3_O_4_	3.0	0.5	– 0.40	425	(41)
NiCo_2_O_4_@Ni foam	3.0 (KOH)	0.4	– 0.60	330	(42)
Pt–Co NS@C sponge	3.0 (KOH)	1.5	– 0.50	353	(43)
NanoAg	1.0	0.125	– 0.15	23.92	(44)
Zn@Pd/CNT	3.0	0.5	– 0.30	835	this work

**Figure 8:**
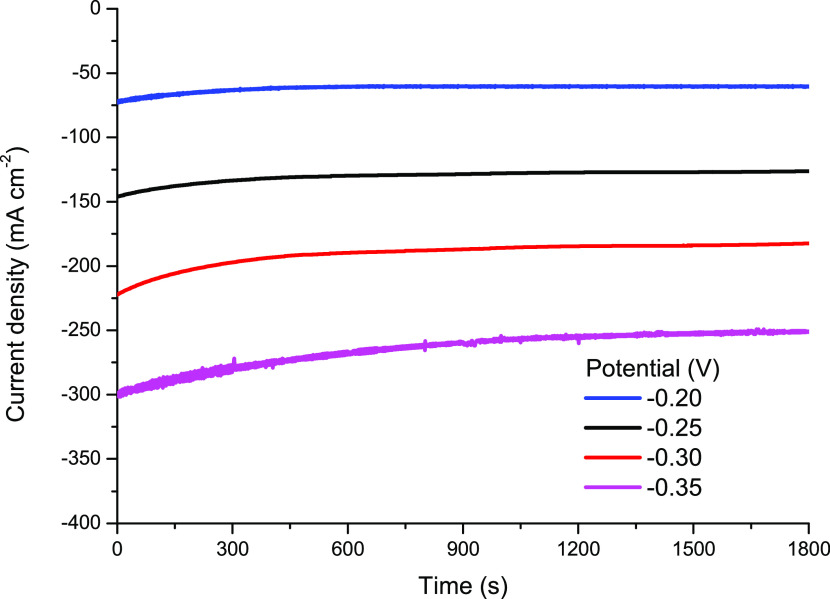
Chronoamperometric curves for H_2_O_2_ reduction on the Zn@Pd/CNT-modified GCEs in 3.0 M NaOH + 0.25 M H_2_O_2_ at different potentials.

Chronoamperometric studies were performed at various applied potentials to further demonstrate the performance of the Zn@Pd/CNT catalyst for H_2_O_2_ electro-reduction. The response of Zn@Pd/CNT-modified GCE at each potential increased significantly and reached a stable value in a short time after the test began as the potential went negative (Figure 8). The CA curves exhibit a gentle fluctuation when the potential is switched to a more negative value, which can be attributed to the disruption of the O_2_ bubbles brought on by H_2_O_2_ breakdown.

**Figure 9:**
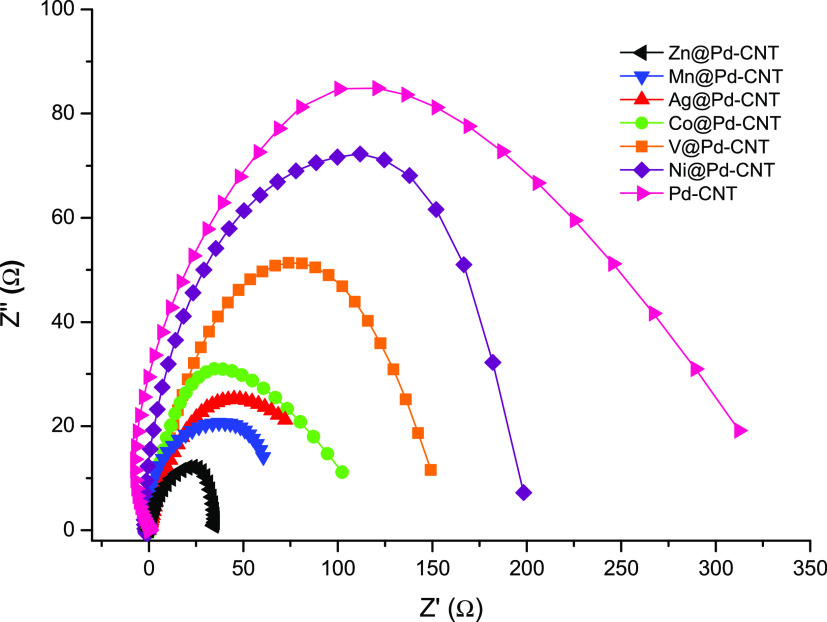
Nyquist plots of H_2_O_2_ electro-reduction on the M@Pd/CNT (M: Zn, Mn, Ag, Co, V, Ni)-modified GCEs at −0.3 V in 3.0 M NaOH+0.25 M H_2_O_2_.

EIS is a commonly used technique for exploring the charge transfer characteristics of the electrodes. At this point of the study, EIS was applied to explore the charge transfer resistance of electrodes modified with different catalysts. The typical Nyquist diagrams of M@Pd/CNT (M: Zn, Mn, Ag, Co, V, Ni)-modified GCEs obtained at −0.3 V are given in Figure 9. A single semicircle that is associated with the charge transfer resistance (Rct) at the solid–electrolyte interface can be seen in the high-frequency region. The Rct values of M@Pd/CNT (M: Zn, Mn, Ag, Co, V, Ni) and Pd/CNT catalysts are about 35, 73, 105, 157 and 200, 350 Ω, respectively. It appears that Zn@Pd/CNT has the smallest resistance at the catalyst–electrolyte interface, indicating that it would be better suited to serve as the cathode catalyst for a H_2_O_2_ fuel cell.

## 4 Conclusions

Palladium-based catalysts were prepared on CNT by the sodium borahydrate reduction method. M@Pd/CNT (M: Zn, Mn, Ag, Co, V, Ni) electrocatalysts’ activities toward H_2_O_2_ reduction in alkaline solution were evaluated using different electrochemical methods. The Zn@Pd/CNT catalyst has a current density of 273.2 mA cm^–2^, which is 3.95 times higher than that of Pd/CNT catalyst-modified electrodes with respect to the CV results. The findings of CA curves further demonstrated that Zn@Pd/CNT has the highest steady-state current density for the H_2_O_2_ electro-reduction process among the synthesized electrocatalysts. Additionally, because Zn@Pd/CNT showed the lowest charge transfer resistance (35 Ω) compared to the other electrocatalysts, the findings of the EIS spectra confirmed the results of CV and CA. The results showed that Zn@Pd/CNT-modified GCE for the H_2_O_2_ electro-reduction reaction had excellent catalytic activity and stability, and it could be used as a catalyst.
